# Supplementary figures and images for: The Relationship Between Bacterial Flora in Saliva and Esophageal Mucus and Endoscopic Severity in Patients with Eosinophilic Esophagitis
Source: Int J Mol Sci. 2025 Mar 26;26(7):3026. doi: 10.3390/ijms26073026 (PMC11989152; doi:10.3390/ijms26073026)

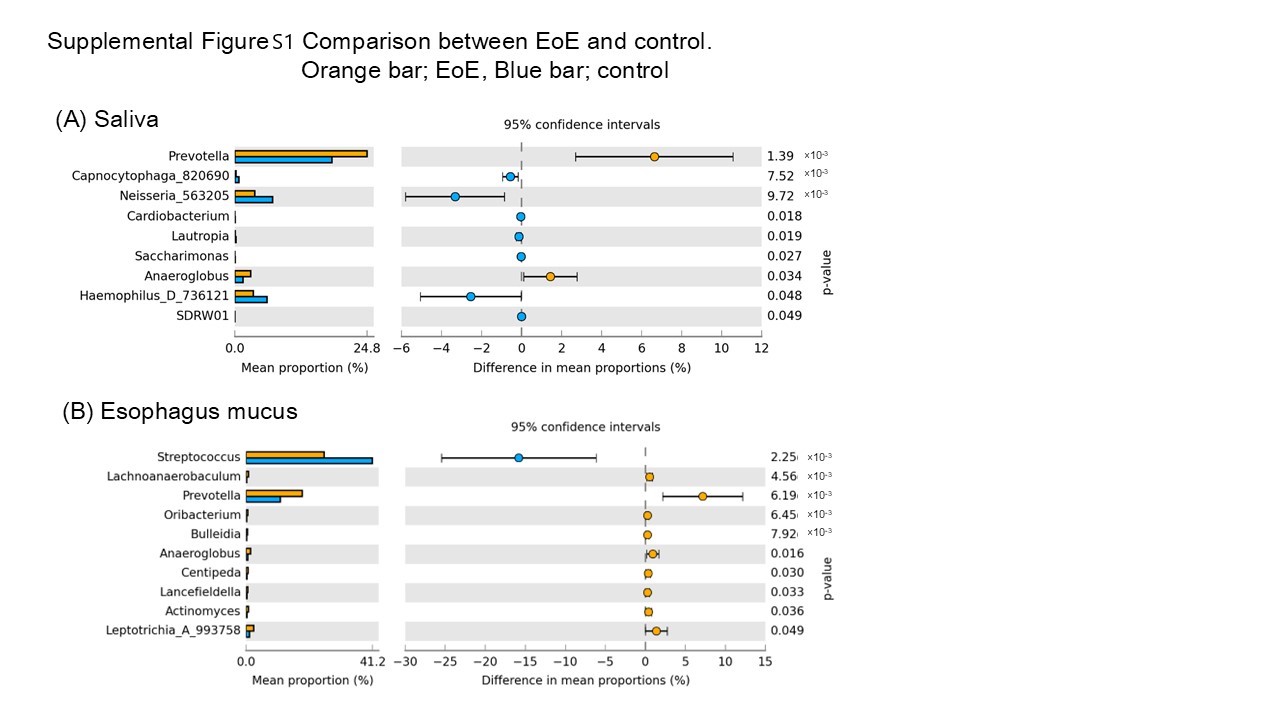

Supplement: Supplementary file 1 [file ijms-26-03026-s001.zip › ijms-3487799-supplementary.jpg]
